# Characterization of tumour-like inclusions in breast-mimicking phantoms using ultrasound optical tomography

**DOI:** 10.1038/s41598-025-18902-1

**Published:** 2025-09-15

**Authors:** Akvilė Zabiliūtė-Karaliūnė, Eglė Bukartė, Maria Ruchkina, Adam Kinos, Alexander Bengtsson, David Hill, Nina Reistad, Lars Rippe, Johannes Swartling, Gabriella Dravecz, Krisztián Lengyel, Magnus Dustler, Nadia Chaudhry, Predrag R. Bakic, Sophia Zackrisson, Stefan Kröll

**Affiliations:** 1 Department of Physics, Division of Atomic Physics, Lund University, Lund, Sweden; 2 Deep Light Vision AB, Lund, Sweden; 3 Institute for Solid State Physics and Optics, HUN-REN Wigner Research Centre for Physics, Budapest, Hungary; 4 Department of Translational Medicine, Radiology Diagnostics, Lund University, Lund, Sweden; 5 Department of Imaging and Physiology, Skåne University Hospital, Malmö, Sweden; 6 Unilabs Breast Center, Malmö, Sweden

**Keywords:** Engineering, Medical research, Optics and photonics, Physics

## Abstract

Ultrasound Optical Tomography (UOT) combines the high-resolution imaging capability of ultrasound with measurements of light absorption and scattering properties of human tissue. In this work, UOT working at 794.2 nm wavelength and equipped with a spectral hole burning filter was used to image through 5 cm thick tissue phantoms with embedded cubic 12 × 12 × 12 mm^3^ inclusions at a 2.5 cm depth. In contrast to earlier UOT works at tissue-relevant wavelengths, these inclusions have orders of magnitude lower absorption and actually mimic the optical properties of human breast tissue with various lesions. Phantoms with inclusions of increasing reduced scattering (inclusions 9.37–14.1 cm^−1^, background 7.83 and 8.5 cm^-1^) and absorption (inclusions 0.061–0.086 cm^−1^, background 0.044 and 0.045 cm^-1^) coefficients were investigated. In the UOT images, the contrast-to-noise ratios varied between 3.87 ± 1.71 and 6.25 ± 2.96, and the inclusions could be easily identified by eye. This indicates that the UOT technique has the potential for spatially resolved imaging and optical data acquisition through at least 5 cm of soft tissue. Our findings suggest that UOT equipped with a spectral hole burning filter is a promising technique for breast tumour imaging as well as qualitative and quantitative characterization of their optical properties.

**Supplementary Information:**

The online version contains supplementary material available at 10.1038/s41598-025-18902-1.

## Introduction

Breast cancer is the most common cancer among women, with over 2 million new cases globally every year^1^. Early detection can significantly reduce the mortality from breast cancer, making effective cancer detection techniques essential for timely treatment. The current standard for early breast cancer diagnosis, i.e. secondary prevention, in most countries is screening mammography, the X-ray imaging of the breast. However, mammography has notable limitations, including the inability to detect cancers that are not visible due to overlapping tissue and the occurrence of false positive findings^2^. An alternative imaging modality is breast ultrasound (US), which uses acoustic waves to visualize breast tissue^3^. US is a safe (as compared to X-ray mammography) and low-cost technique. Still, the specificity (the proportion of actually negative cases identified as negative) of US imaging is insufficient, and a needle biopsy is required to differentiate benign from malignant findings through histopathological diagnosis^4^.

In view of this, optical imaging emerges as a promising complementary tool for medical diagnostics, providing non-invasive, functional information about the tissue. For this approach, light in the so-called tissue optical window (~ 650–900 nm) is used as it is the least absorbed in human tissue^5,6^. It is known that light scattering and absorption properties differ between healthy and cancerous (breast) tissues^7–11^. The ability to distinguish between regions with different optical properties can help to locate, evaluate, and define cancerous lesions and differentiate between malignant and benign ones^10^. Considering and capturing changes in the ratio between oxygenated and deoxygenated haemoglobin, which is highly stable in normal conditions, may indicate an underlying abnormality, such as hypoxia caused by a tumour^12^. Due to these reasons, optical imaging has received much attention recently^13–15^.

Although light is highly scattered in biological tissue, many optical imaging techniques have been developed that overcome this problem^16^. For example, optical coherence tomography (OCT) is an interferometric technique which uses depth-wise reflections to make the cross-sectional images of the sample^17^. Although resolution of OCT can reach sub-µm range, its penetration depth is only up to 1 mm^16^. Another well-known technique is diffuse optical tomography (DOT) which is already applied in some areas of medicine^18^. In DOT light propagates and scatters in the tissue while travelling from the source to the detector. By combining several sources or detectors and by changing the distance between them it is possible to calculate light scattering and absorption properties of the medium and even extract information about blood oxygenation^18^. However, the main drawback of this technique is a rapid decrease of resolution with the imaging depth^19^. Larger depths can be reached using photoacoustic imaging (PAI), where light-generated acoustic waves are measured, and signals from depths up to a few cm have been reported^14^. A few clinical trials used differences in oxy- and deoxyhaemoglobin as a baseline for the photoacoustic signal in breast lesion characterization using two-wavelength sampling^20–22^. A deeper imaging depth than previously shown would open up for both improved breast imaging and applications of optical imaging in other organs.

A promising deep tissue optical imaging technique is ultrasound optical tomography (UOT) as it could potentially study the optical properties of human tissues at a depth of up to several centimetres, considerably deeper than other similar techniques^15,23^. Moreover, our previous theoretical study shows that UOT is likely to reach deeper in the tissue and attain larger CNR values if compared to PAI^24^. UOT combines ultrasound and light to obtain deep tissue images with ultrasound resolution. It is based on the acousto-optic effect, where the carrier light is frequency-shifted (tagged) by the US and thus can be traced even in a high scattering media. The concept of UOT was presented in 1993^23^ and it has been developed ever since^15,25^. Recent application in glucose monitoring also proves that UOT has the potential in molecular sensing^26^. The most challenging part of UOT is the separation of the tagged photons from the carrier ones as the difference of their frequencies is only in the order of MHz. Several approaches that solve this problem have been investigated: photorefractive detection which employs a photorefractive material to detect tagged light, single-shot off-axis holography which is based on the interference pattern between the signal and the reference fields, speckle contrast which analyses the speckle contrast change due to the interaction with the US field, and spectral hole burning, which is further discussed in this research (SHB). They all have their own advantages but according to simulations, the best Contrast-to-Noise Ratio (CNR) value is obtained through the use of the SHB detection method^27,28^. Moreover, as shown by Li et al. who introduced UOT using SHB filters at 793 nm wavelength already in the year 2008, this technique is characterized by a large etendue (the product between the detector area and the acceptance solid angle) and is insensitive to speckle decorrelation^29^.

In our previous research, we presented UOT equipped with a Pr^3+^:Y_2_SiO_5_ SHB crystal filter^30^. However, this crystal works for a wavelength of around 606 nm, which is outside the biological optical window, and therefore its applications to UOT are limited. Several research groups have already demonstrated UOT with SHB filters working in the tissue optical window: Tm^3+^:YAG crystals were used to detect US tagged light^31,32^, Li et al. have demonstrated UOT tomograms imaging India ink absorbers in 10 mm thick tissue phantoms^29^, and Xu et al. demonstrated images with dark (absorption coefficient, $\mu_{a,c} = 100{\text{ cm}}^{ - 1}$) absorbers in 32 mm thick chicken breasts^33^. Although the inclusions here were imaged with a high resolution and even fine details could be distinguished, the absorbers had much higher absorption properties compared to the real-life tumours in human tissue. For instance, the absorption coefficient of breast tumours is around $\mu_{a,c} = 0.085 \pm 0.017{\text{ cm}}^{ - 1}$^7–9^, which is more than 2000 times smaller than that of the inclusions in Ref^33^.

In this work, we apply UOT equipped with a Tm^3+^:LiNbO_3_ crystal SHB filter working at ~ 790 nm, i.e. in the biological optical window range, to image through realistic 5 cm thick agar-intralipid breast tissue phantoms with cubic 12 × 12 × 12 mm^3^ tumour mimicking inclusions at 2.5 cm depth. The absorption and reduced scattering coefficients of the phantoms and inclusions are chosen to be similar to those of real human tissues and tumours, respectively, presented in the literature^7–9^. In total, six phantoms are imaged: three with the inclusions of increasing reduced scattering and fixed absorption, and three with inclusions of increasing absorption and fixed reduced scattering. Alongside the images, the CNR values are calculated and discussed. Our findings suggest that UOT equipped with a SHB filter is a promising technique for tumour imaging and for qualitative as well as quantitative characterization of their optical properties.

## Materials and methods

### Ultrasound optical tomography

**Fig. 1:**
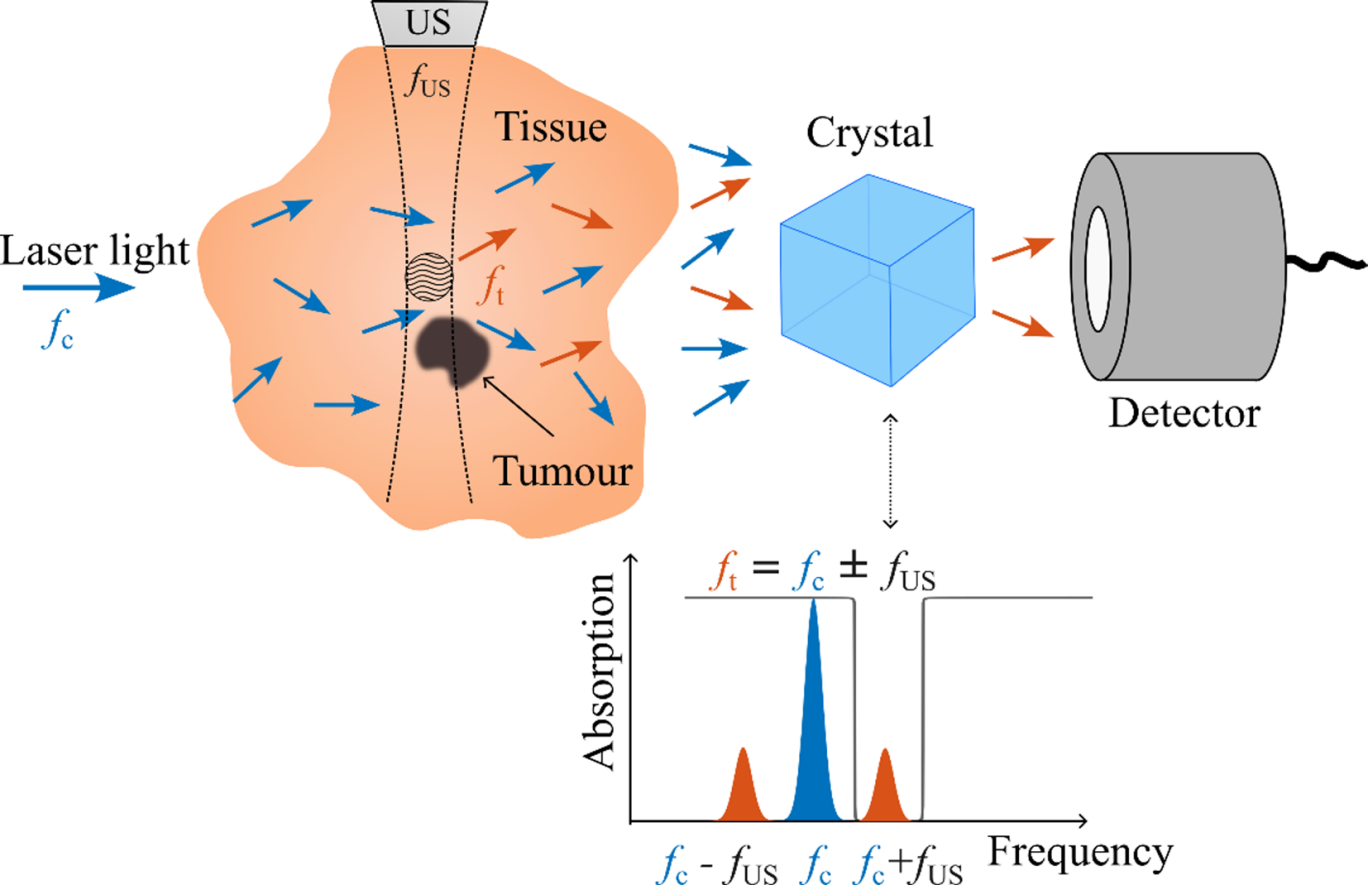
Illustration of the UOT technique. Pulsed US waves of frequency *f*_US_ and pulses of laser light frequency, *f*_*c*_ (blue arrows), are simultaneously delivered into the target tissue. Photons interacting with the US pulse (schematically represented by the encircled area slightly above the tumour) are frequency-shifted (tagged) to the new frequencies *f*_t_ = *f*_c_ ± *f*_US_ (red). The light exiting the tissue is collected and directed towards the SHB filter crystal. The crystal absorbs the carrier light, but transmits the tagged photons in one of the sidebands.

UOT is a sophisticated technique for imaging deep soft tissue, and its overview is presented in Fig. 1. Pulsed US waves (frequency *f*_US_) and pulses of optical light (*carrier* photons with frequency $f_{c}$, blue) are simultaneously sent into the tissue of interest. Carrier photons passing the US pulse interact with it and get frequency shifted i.e. tagged ($f_{t}$, red) by the US-frequency due to the acousto-optic effect ($f_{t} = f_{c} \pm f_{US}$). As the fraction of tagged photons at the tissue (phantom) output typically is relatively low (~ 1%) and the frequency shift is in the order of few MHz, which, compared to the carrier frequency in hundreds of THz, is incredibly small, it is challenging to filter out the photons of interest. UOT using the SHB method separates the tagged light from the carrier light by using a narrowband optical filter formed in a rare-earth-ion-doped crystal using optical pumping and SHB techniques^27,29^.

**Fig. 2:**
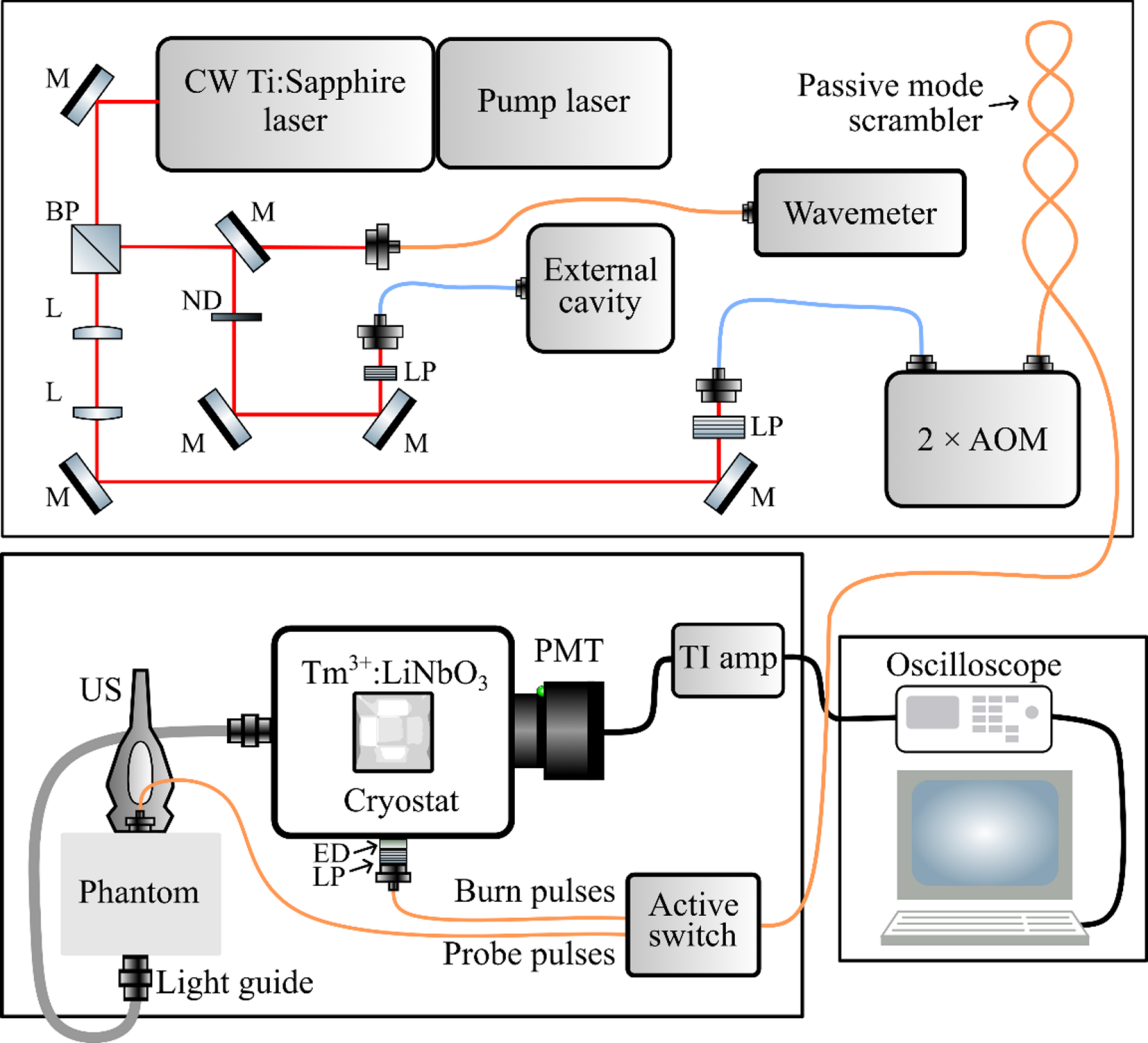
Schematic representation of the UOT experimental setup. A laser system composed of a pump laser and a narrow-band tuneable Ti:Sapphire laser, which is locked to an external cavity, produces a continuous laser output at 794.2 nm (377.4 THz). Optical pulse shaping is performed using a double-pass acousto-optic modulator (2 × AOM), and pulse switching between the burn pulses (to create the spectral hole-burning filter) and probe pulses (to be sent into the phantom) is performed by an active switch. The narrowband optical filter is created inside a stoichiometric Tm^3+^:LiNbO_3_ crystal, placed inside the cryostat, and cooled to 3 K. Ultrasound-tagged photons, shifted by 6 MHz, are transmitted through the filter and collected using a photomultiplier tube (PMT). The signal is then amplified with a transimpedance amplifier (TI amp) and recorded with an oscilloscope. Other abbreviations used in the figure are: M—mirror, BP—beam pick-off, L—lens, ND—neutral density filter, LP—linear polarizer, and ED—(square) engineered diffuser. Blue and orange lines are polarization maintaining and multimode fibres, respectively.

The schematic representation of the experimental UOT setup in transmission mode is presented in Fig. 2. A diode-pumped solid-state laser (Sprout-G, Lighthouse Photonics) pumped a tuneable continuous-wave Ti: Sapphire laser (SolsTiS-SRX-XF, M squared) to produce a narrow linewidth laser output at 794.2 nm (377.4 THz), which lies within the tissue optical window. For additional stability, the laser mode was locked to the external cavity (ORS-Cubic-Cavity, MenloSystems), and the set wavelength was monitored via the wavemeter (WS-61R HighFinesse, Ångstrom). Optical pulse shaping was performed using a lab-built compactly designed setup (2 × AOM) where a single acousto-optic modulator (AOMO 3100–125 Gooch & Honsego) is operated in a double pass configuration^34^. Switching between the burning pulses (to create the SHB filter) and the probe pulses (to be sent into the phantom) was conducted using an active fibre 1 × 2 switch (Agiltron FFLS, 780 nm). The peak power of light incident on the sample was 170 mW which corresponds to 2.36 mW average power in the pulsed mode. Having the spot size of 7.96 × 10^–3^ cm^2^ yelded to 296.4 mW/cm^2^ power density which is below the medical safety limit (309 mW/cm^2^ for 794.2 nm)^35^. If the laser power is to be increased, the power density can be reduced by enlarging the spot size which is achieved by adjusting the distance between the input fibre and the phantom.

An US system (Verasonics Vantage 32LE) with a linear array transducer (Philips, L7-4) generated US pulses with a 6 MHz frequency. The transducer array had a length of 42 mm and consisted of 128 piezoelectric elements that could be individually activated. For the measurements, the transducer was programmed to activate overlapping groups of 12 elements to generate 21 parallel sequential unfocused US pulses along the whole length of the array. This separated two consecutive US pulses by 2 mm along the horizontal UOT image axis, and each pulse had a duration of ~ 1.5 µs. As the phantom composition was mostly water (> 95%), the acoustic velocity of the US pulse was assumed to be the same as it is in water (1498 m/s). During the propagation of each US pulse, optical laser pulses were sent into the sample. The time spacing between optical laser pulses was set to 1.35 µs, resulting in a 2 mm vertical distance between consecutive measurements. The first 5 mm of the phantom surface was not imaged due to significant sound pressure fluctuations in the near field of the US transducer.

The narrowband optical filter for tagged photons was created inside a stoichiometric Tm^3+^:LiNbO_3_ crystal, which was grown by the high temperature top seeded solution growth method from K_2_O containing flux with 0.05 mol% nominal Tm^3+^ doping concentration^36^. X-ray oriented optical sample with dimensions of 5 × 5 × 5 mm^3^ was cut and polished for measurements. The crystal was placed inside a custom-made closed-cycle helium cryostat from *MyCryoFirm* and kept at 3 K. Additional optics inside the cryostat can be found in Fig. 6.16 in Ref^37^. To form a spectral hole at the frequency of the tagged first sideband photons $(f_{t} = f_{c} + f_{US}$), the frequencies between the burn and the probe pulses differed by 6 MHz. The filter had a 23 dB relative attenuation for the carrier photons. The width and lifetime of the filter were 5 MHz and 0.171 s, respectively.

A limitation of the Tm^3+^:LiNbO_3_ crystal filter was the existence of a crystal field Stark level positioned just 8 cm^-1^ above the ground state. After absorbing the carrier photons, some atoms decayed to this Stark level. The wavelength of this fluorescence was shifted by ~ 0.5 nm from the tagged light and was far outside of the absorption range of the hole-burning filter, while still being too close in wavelength to be blocked by a conventional dichroic filter. Therefore, after each train of laser pulses, before sending a new US pulse to the phantom, a waiting time of 1 ms was added allowing the built-up fluorescence signal to decay. This introduced significant delays and prolonged the experimental time. Only six consecutive measurements could be performed before the filter hole decayed to the extent that it needed to be recreated.

Tagged photons, transmitted through the filter, were collected with a photomultiplier tube (PMT, Hamamatsu H11526-20). The signal was then amplified with a transimpedance amplifier (Femto, DHPCA-100) and recorded with an oscilloscope (Le Croy, Waverunner HRO 66Zi). One measurement of a single phantom took 520 s (almost 9 min) and in total contained 6000 averages. Schematic representation of image acquisition steps is presented in Supplementary Fig. S1. The long experimental time was mainly caused by the waiting time for the fluorescence to decay. In principle, if the fluorescence was eliminated, the experimental time could have been reduced by roughly 50 times. This could be done by replacing the Tm:LiNbO_3_ crystal by a crystal where the lowest ground state crystal field Stark levels is spectrally well separated from the other crystal field Stark levels.

### Physical breast tissue phantom design and fabrication

Tissue-mimicking agar-intralipid phantoms were prepared using a recipe based on Refs.^38,39^. Deionized water was mixed with agar powder (A7921, Sigma-Aldrich) with an agar-to-water ratio of 1 g to 100 ml. The mixture was then heated up to 95 °C using a hot plate. The temperature was monitored with a digital thermometer, and the mixture was stirred with a magnetic stirrer throughout the phantom-making process. The agar-water mixture was maintained at 95 °C for one hour and then left to cool to 45 °C. At this temperature, India ink from a stock solution (ink-to-water ratio of 1:200) and intralipid emulsion (I141, Sigma-Aldrich) were added to the mixture. The ink and intralipid emulsion volumes defined the absorption and reduced scattering coefficients of the phantoms and their inclusions, respectively. For instance, for the healthy tissue mimicking phantom in Batch 1 (Table 1), 4.1 vol% of intralipid and 0.019 vol% of ink were added. For the tumour-mimicking inclusion with the highest absorption, the corresponding amounts were 5 vol% and 0.028 vol%, respectively. These amounts were added gradually, while monitoring the optical properties of the liquid phantoms using optical time-of-flight spectroscopy^40^. After adding appropriate volumes of ink solution and intralipid emulsion, the mixture was poured into cubic (dimensions of 65 × 65 × 65 mm^3^) silicone moulds and left in a fridge overnight to solidify. After the solidification, the absorption and scattering properties were measured again. The other batch was prepared in a similar manner.

**Table 1:** Optical properties of agar-intralipid phantoms.

Healthy tissue	$\mu_{a,c}$, cm^−1^	$\mu_{a,c}$/$\mu_{a,h}$	$\mu{\prime}_{s,c}$, cm^−1^	$\mu_{s,c}{\prime}$/$\mu{\prime}_{s,h}$
Batch 1 $\mu_{a,h}$ = 0.045 cm^−1^ $\mu{\prime}_{s,h}$ = 8.5 cm^−1^	0.061	**1.36**	12.7	1.49
	0.071	**1.58**	12.7	1.49
	0.086	**1.91**	12.9	1.52
Batch 2 $\mu_{a,h}$ = 0.044 cm^−1^ $\mu{\prime}_{s,h}$ = 7.83 cm^−1^	0.069	1.57	9.37	**1.19**
	0.071	1.61	11.9	**1.52**
	0.076	1.72	14.1	**1.80**

Absorption and reduced scattering coefficients of the phantoms mimicking healthy breast tissue ($\mu_{a,h}$ and $\mu{\prime}_{s,h}$) are presented in the first column. The absorption and reduced scattering coefficients for the inclusions mimicking cancerous tissue ($\mu_{a,c}$ and $\mu{\prime}_{s,c}$) are given in the second and fourth columns, respectively. The coefficient ratios are presented in the third and fifth columns. The optical property that was varied between the different phantoms in the same batch is written in bold.

Significant values are in [bold].

As the first step, phantoms mimicking the properties of cancerous tissues were prepared. 12 × 12 × 12 mm^3^ cube inclusions, cut from these phantoms, were placed on threads inside the silicone moulds such that their centres would be approximately in the middle (~ 2.5 cm) of the final phantoms. A liquid phantom mixture, corresponding to healthy tissue, was then poured into the silicone moulds around the inclusions to form 50 mm thick phantoms with inclusions and left to solidify. Threads holding inclusions were removed after solidification. An additional phantom without an inclusion (blank) was created using the same mixture and later measured to be used as a reference for the UOT signal. After solidification, all phantoms were examined with US to confirm the correct positions of the inclusions.

As summarized in Table 1, two different batches of phantoms were made. Both batches had three phantoms with inclusions and one phantom without an inclusion. The “healthy tissue” was made of the same material in all four phantoms in the same batch. These experiments aimed to probe the technique’s capabilities for detecting inclusions with varying absorption and scattering properties. Both batches, therefore, had one property that was varied while the other remained constant. In Batch 1, the absorption coefficient for the inclusions was varied, aiming for the inclusions to be 1.3, 1.6, and 2 times more absorbing than the surrounding medium for healthy tissue. The ratio of the reduced scattering coefficients was aimed to be 1.6. In Batch 2 the reduced scattering coefficient was varied, with the objective for inclusions to scatter 1.3, 1.6, and 2 times more than the surroundings. The ratio of the absorption coefficients for this batch was here aimed to be constant at 1.6.

Table 1 gives the obtained absorption and reduced scattering coefficients for all the phantoms and their inclusions as well as the ratios between the coefficients. As the coefficient values measured with time-of-flight spectroscopy after solidification increased by 7.11 ± 4.67% for absorption and 36.53 ± 10.05% for reduced scattering coefficients compared to before solidification, obtaining the exact planned ratios (1.3, 1.6, and 2) was challenging. As summarized in Table 1, for Batch 1, the ratios of absorption coefficients ($\mu_{a,c}$/$\mu_{a,h}$) were obtained as follows: 1.36, 1.58, and 1.91, while the ratio of the reduced scattering coefficients ($\mu{\prime}_{s,c}$/$\mu{\prime}_{s,h}$) stayed at around 1.5. For Batch 2, the ratios of the reduced scattering coefficients were 1.19, 1.52, and 1.8. Although the ratio of the absorption coefficients was attempted to be kept constant at 1.6, it changed to 1.57, 1.61, and 1.72 with increasing scattering. The increase of the absorption coefficient was caused by the addition of intralipid, since it increases not only the scattering but also the absorption properties of the mixture.

It is important to note that in this experiment, the absorption and scattering properties of the phantoms are very close to those of real human tissues. For reference, the optical properties of healthy and cancerous breast tissues, as found in the literature, are: $\mu_{a,h} = 0.041 \pm 0.025{\text{ cm}}^{ - 1}$ and $\mu{\prime}_{s,h} = 8.5 \pm 2.1{\text{ cm}}^{ - 1}$ for healthy breast tissue and $\mu_{a,c} = 0.085 \pm 0.017{\text{ cm}}^{ - 1}$ and $\mu{\prime}_{s,c} = 12.7 \pm 0.3{\text{ cm}}^{ - 1}$ for cancerous tissue^7,8^. Based on these numbers, the real-life case would be best represented by the phantom of the strongest absorbing inclusion in Batch 1.

To evaluate errors introduced by the time-of-flight equipment, absorption and reduced scattering coefficients were measured at three different locations on three blank phantoms. The standard deviation of the three values from each phantom was calculated, and the percentage change from the mean value was used as the relative error for the absorption and reduced scattering coefficients. The final error for each coefficient was the average of calculated relative errors from the three phantoms. These values were 3.4% for absorption and 2.8% for reduced scattering coefficients. To evaluate the errors in the ratios $\mu_{a,c}$/$\mu_{a,h}$ and $\mu{\prime}_{s,c}$/$\mu{\prime}_{s,h}$, the two relative errors were added in quadrature, resulting in the errors of ~ 5% and ~ 4%, respectively.

**Fig. 3:**
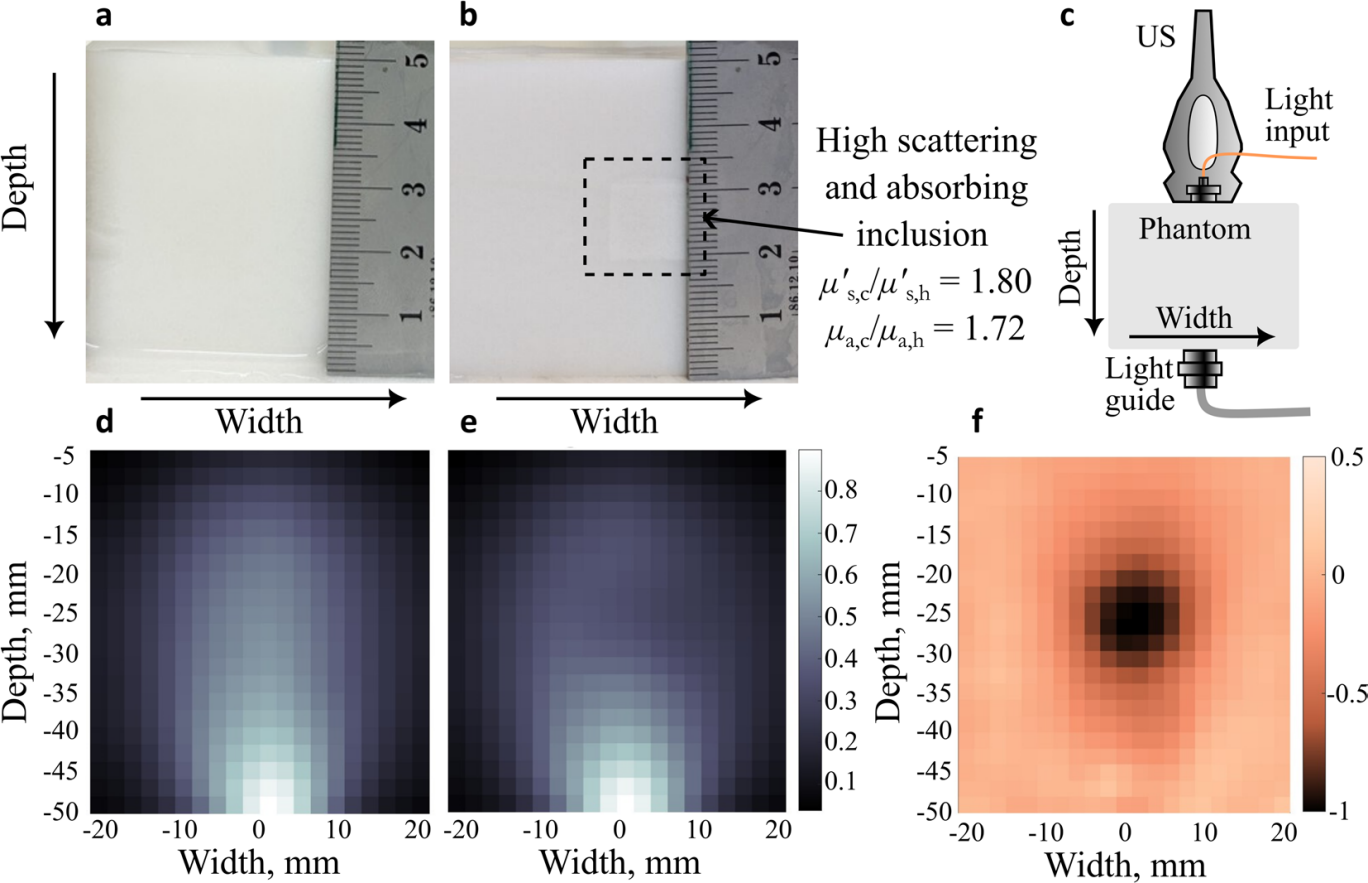
The process of making a UOT image of the phantom with an inclusion. **a** and **b** show the photographs of cross-sections of a blank phantom and a phantom with a 12 × 12 × 12 mm^3^ size, high scattering ($\mu{\prime}_{s,c}$/$\mu{\prime}_{s,h} = 1.8$, $\mu_{a,c}$/$\mu_{a,h} = 1.72$) cancer mimicking inclusion (marked with a dashed square), respectively; **c** the placement of the phantom with respect to the transducer, light input and output, the depth arrow points towards the increasing depth and the width arrow shows the direction of US pulse activation; **d** and **e** show two normalized UOT signals recorded from the phantoms in **a** and **b**, respectively; **f** a UOT image obtained by subtracting the two normalized signals as in Eq. (1).

Photographs of one of the produced blank phantoms and a cross section of a phantom with an absorbing and more strongly scattering 12 × 12 × 12 mm^3^ size inclusion (Batch 2, $\mu{\prime}_{s,c}$/$\mu{\prime}_{s,h} = 1.8$). are presented in Fig. 3a and b, respectively. The phantom in Fig. 3b is cut right through the inclusion, which is surrounded by a dashed square. Although the inclusion has a larger absorption than the surroundings, it appears as a lighter square when compared to the surroundings. This is due to its larger reduced scattering coefficient, which causes the material to backscatter (reflect) more light and thus appear lighter.

### Construction of a UOT image

Figure 3c shows the positions of the US probe and the light input and output relative to the phantom during the measurements. Figure 3d and e show UOT recordings of the blank phantom and the phantom with a high scattering inclusion (Batch 2, $\mu{\prime}_{s,c}$/$\mu{\prime}_{s,h} = 1.8$), respectively. These two recordings were used to construct a single UOT image shown in Fig. 3f. Before forming the image, each recording was normalized to a maximum signal of 1. The recordings represent the normalized tagged light from the US-defined volume (within 2 mm radius from the US position^41^) in the phantom. We can see in Fig. 3d and e, that in our case, the maximum signal intensity is recorded when the ultrasound pulse is just above the signal collecting lightguide located in the centre at the bottom of the phantom. This intensity distribution has a non-trivial dependence of the position of the light input and the lightguide, absorption and reflection at the walls, the absorption and scattering coefficients of the sample and even the positioning of the US transducer and the strength of the US. Similar recordings were also obtained for all phantoms studied in this paper.

Each UOT image consists of 21 × 22 pixels, each of 2 × 2 mm^2^ size. As discussed in Sect. 2.1 the horizontal axis (21 pixels) was defined by the length of the US transducer and the grouping of the piezoelectric elements, and the vertical axis (22 pixels) depended on the thickness of the tissue-mimicking phantoms and the length (duration) of the laser pulses.

The UOT image, *S*, was then obtained by subtracting the normalised signal recorded from the blank phantom, *S*^*blank*^, from the signal of the phantom with an inclusion, *S*^*inclusion*^. The pixel intensity value in each pixel of the UOT image, *i*, could then be expressed as follows:

1 $$S_{i} = S_{i}^{inclusion} - S_{i}^{blank} .$$

As the signal intensity at the inclusion position is lower than the corresponding one from the blank phantom, the resulting pixel value in the UOT image at the inclusion position is negative. The UOT images were plotted using our defined colour scale, which was created by interpolating between five selected colours. Each colour’s RGB values are presented in the Supplementary Table S1. All UOT images were smoothed using a Gaussian filter function *imgaussfilt* in Matlab^42^, with sigma = 0.8 corresponding to a FWHM of ~ 1.9 pixels. The resulting UOT image is presented in Fig. 3f. Generally, the resolution of the system primarily should be defined by the US pulse, i.e. ~ 4 mm. However, in this case it is lower due to the smoothing and interpolation applied during image analysis. By comparing Fig. 3b and f, we observe that although the difference between the inclusion and its surroundings is not pronounced in the photograph, it is clearly visible in the UOT image. This is the case although the picture in Fig. 3b is taken after cutting through the phantom while the UOT signal in Fig. 3f is recorded when the inclusion is covered by 2 cm background phantom material.

### Contrast-to-noise ratios

Contrast-to-noise ratio (CNR) is an essential measure for the evaluation of the technique’s ability to detect small changes in the signal. In our case, the CNR was defined as in Ref.^43^:

2 $$CNR = \frac{{\left| {\mu_{inclusion} - \mu_{background} } \right|}}{{\sigma_{noise} }}.$$

Here, $\mu_{inclusion}$ and $\mu_{background}$ are the average pixel values from two regions of interest (ROI) of the UOT image, one for the inclusion and one for the background, respectively. The *σ*_*noise*_ is the standard deviation of the pixel values in the background region only. CNR = 0 means that there are no differences between the inclusion and the background at all. However, in this work, our aim is to achieve CNR > 1 so that it is possible to distinguish contrast variations between the inclusion and the surroundings.

The ROI of the inclusion for CNR calculations was determined after slicing the phantom and examining its cross-section, so it had a matching size and position in UOT image of the actual inclusion. Eight different ROI’s were chosen for the background. All background ROI’s were of the same size as that for the inclusion, and they were positioned around it in the image. The selected ROIs for each UOT image are indicated in Supplementary Fig. S2.

The accuracy of the CNR was evaluated by calculating eight CNRs from each UOT image—one for each background ROI. The mean and standard deviation of these values were later used as an averaged CNR or error value, respectively.

## Results and discussion

**Fig. 4:**
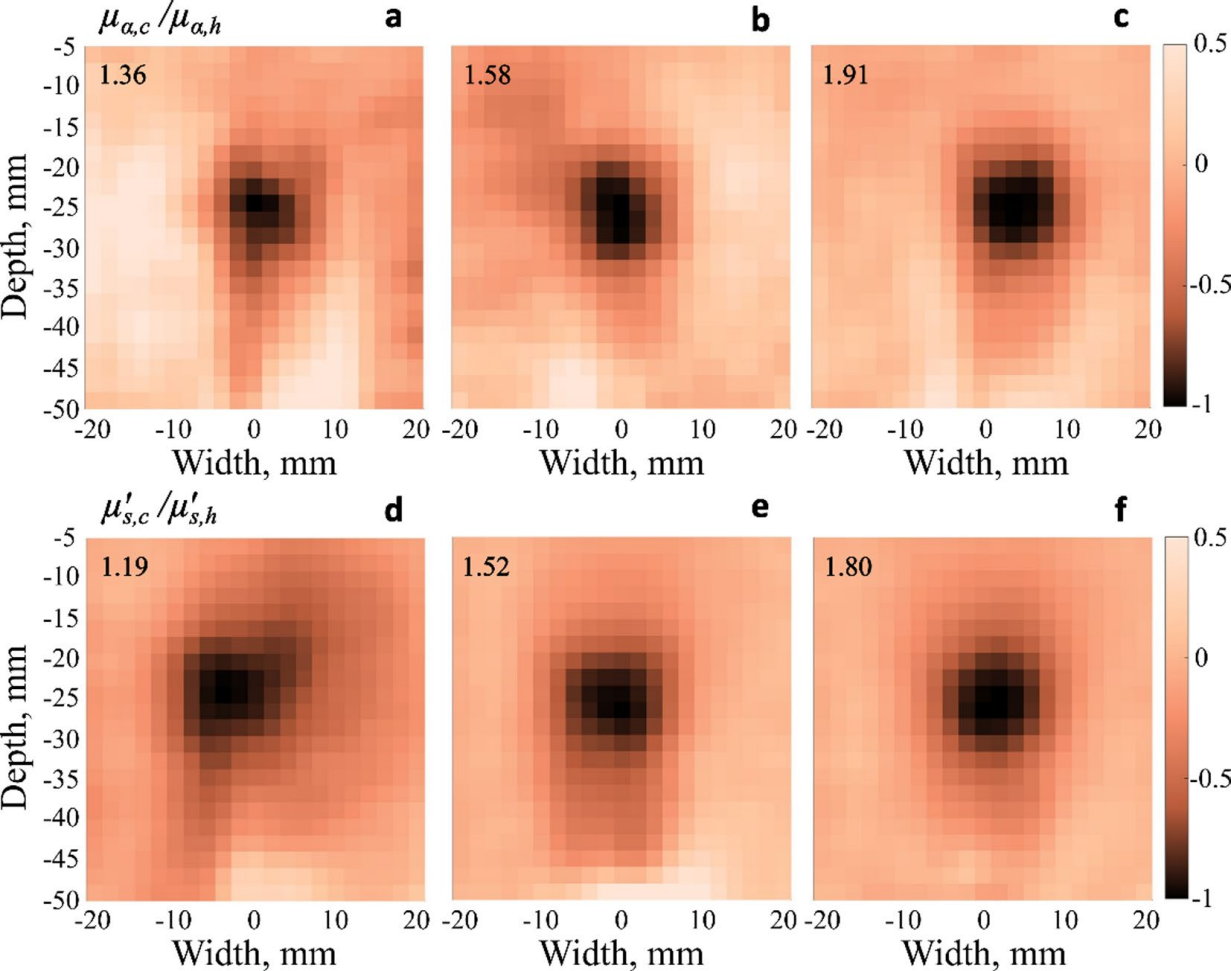
UOT images of 50 mm thick agar-intralipid phantoms with tumour mimicking inclusions. **a–c** show the images with increasing inclusion absorption; **d–f** show the images with increasing inclusion reduced scattering. Absorption and reduced scattering coefficient ratios between the inclusion and the background ($\mu_{a,c}$/$\mu_{a,h}$ and $\mu{\prime}_{s,c}$/$\mu{\prime}_{s,h}$, respectively) are indicated in each subfigure and also summarized in Table 1. In each image, all pixels are divided by the absolute value of the pixel in the image with the most negative value. All images are consequently normalized such that the lowest pixel value is − 1 and then plotted on a colour scale. All images are 42 × 45 mm^2^.

Figure 4 presents UOT images of the six tissue-mimicking phantoms from the two batches with tumour mimicking inclusions having different optical properties. The values of the light absorption and reduced scattering ratios are presented in the corresponding figures and Table 1. All images were normalized to a minimum value of − 1 and plotted to the same colour scale. The US images of all the phantoms are presented in Supplementary Fig. S3. By comparing the UOT and US images, it is clear that in this case, the UOT technique can visualize the inclusions. Characterizing tumours with known location is of great clinical importance, as it informs clinical decisions on further treatment, as well as follow-up to evaluate the treatment effects. Also, it is essential to note that the US provides the acoustic information whereas UOT gives the optical information of the samples. For this reason, we believe that US and UOT are not competing imaging techniques but rather complementary. In the phantoms studied in this paper, the optical properties of the “healthy” and “tumour” tissue differ, but their acoustic properties are the same. However, air bubbles at the interface between the “healthy” phantom and the inclusion appear as white lines in Supplementary Fig. S3.

Looking at the UOT image in Fig. 4a, we note that using a fixed reduced scattering ratio between cancerous and healthy phantom regions $\mu{\prime}_{s,c}$/$\mu{\prime}_{s,h} = 1.49$ the inclusion is readily observed even for the smallest absorption coefficient ratio $\mu_{a,c}$/$\mu_{a,h} = 1.36$. Analogous observations are also made from Fig. 4d, where for the absorption coefficient ratio between tumour- and healthy tissue-mimicking phantom regions $\mu_{a,c}$/$\mu_{a,h} = 1.57$ the inclusion is clearly visible for the smallest reduced scattering ratio $\mu{\prime}_{s,c}$/$\mu{\prime}_{s,h} = 1.19$.

**Fig. 5:**
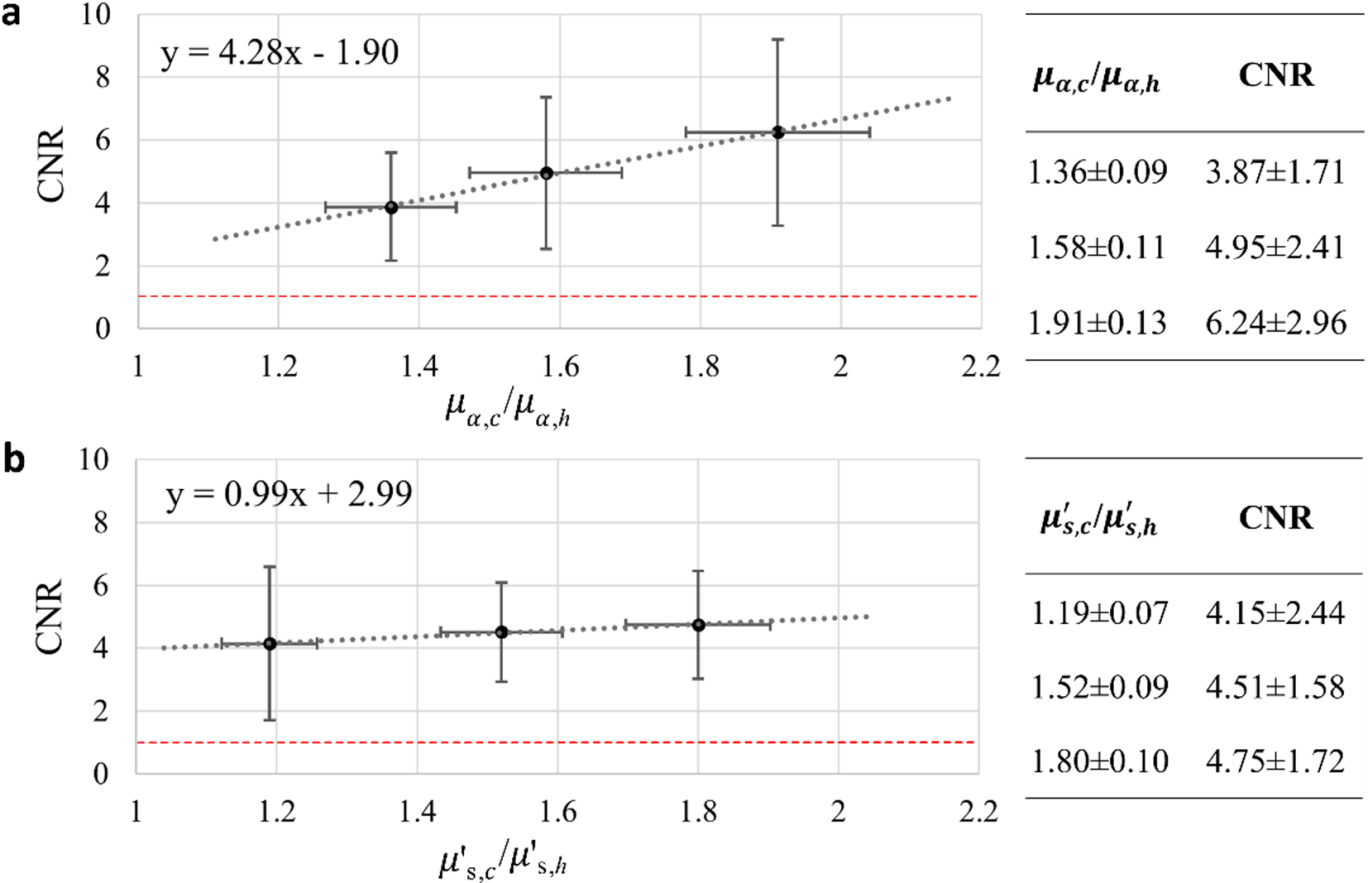
CNR of UOT images of phantoms with different optical properties of inclusions. **a** The CNR dependency on the ratio of absorption coefficients ($\mu_{a,c}$/$\mu_{a,h}$). **b** The CNR dependency on the ratio of reduced scattering coefficients ($\mu{\prime}_{s,c}$/$\mu{\prime}_{s,h}$). The exact absorption and scattering coefficient ratios and CNR values are presented in corresponding tables. Black dashed lines represent the linear fits, red dashed lines indicate CNR = 1.

The quantitative analysis of the UOT images for both batches of tissue mimicking phantoms is presented in Fig. 5. We use CNR as a parameter characterizing the imaging capabilities of the system as it helps us to understand how far are we above the detection limit. In the future development of the system other parameters may turn out to be more useful. If we compare the linear fits of the CNR increase with $\mu_{a,c}$/$\mu_{a,h}$ and $\mu{\prime}_{s,c}$/$\mu{\prime}_{s,h}$, we can see that the difference in absorption coefficient has a more substantial influence. Moreover, in Fig. 5a the CNR increases 50% when the absorption increases by 40%. In column 3 in Table 1 one may note that the absorption in batch 2 is not constant, but increases by about 10% going from the lowest to the highest reduced scattering coefficient. Judging from Fig. 5a, this absorption increase would lead to an 8% increase in CNR. This suggests that most of the increase of the CNR in Fig. 5b actually might not be caused by an increase in reduced scattering coefficient but instead by increased absorption. Intuitively one may think that an increased reduced scattering coefficient would lead to a stronger tagging signal but this does not seem to be supported by the data in Fig. 5b. However, given the considerable uncertainty in the CNR values the validity of the statement of linearity would need to be checked by additional experiments or simulations. The low sensitivity to reduced scattering could have both advantages and disadvantages. Potentially, it might mean more robust quantification of the absorption. On the other hand, it could be an advantage to also be able to see changes in scattering as this also could carry information about the tissue status.

From Eq. 2 it follows that if both the reduced scattering and absorption ratios are equal to 1 (meaning the inclusion absorbs or scatters the same as the surrounding tissue) one could expect CNR = 0. However, we can observe in Fig. 5 a and b that the linear fits do not even fall below CNR = 1 when extrapolated to $\mu_{a,c}$/$\mu_{a,h} = 1$, or $\mu{\prime}_{s,c}$/$\mu{\prime}_{s,h} = 1$. This happens due to the fact that in our case both ratios are never equal to 1. In other words, for the phantom batch with varying absorption coefficient ratio the reduced scattering coefficient ratio is fixed to a value above 1 and vice versa.

Given that it is very easy to spot the inclusions in the images the CNR error bars may seem too large. However, by examining Fig. 4 one can clearly see that the regions representing healthy tissue do not appear homogeneous and often have fairly large lighter and darker areas. This is also validated quantitatively in Supplementary Fig. S2 where CNR values between these areas and the regions of the inclusions, are presented. As we can see the CNR values, depending on the selected background ROI, can vary by more than a factor of 3. This is caused by the relatively strong large-scale variations in the overall background. We believe that the inhomogeneities in the images are mainly caused by the changes in the relative positions of the US transducer with respect to the lightguide (see Fig. 2) when switching between the blank phantom and the corresponding phantom with the inclusion. As the final UOT images are obtained after subtracting the image of the blank phantom from the image of the one with the inclusion, even a slight misalignment made while changing the phantoms becomes visible in the final images. In the future, this issue could be addressed by utilising a more stable and controllable US transducer holder. Simulations can also be important in assessing how misalignment affects data and how it can be corrected. Moreover, they might also be relevant for data interpretation and information extraction. Such issues in in vivo applications could be solved by several different approaches which avoid taking blank images: creating digital blank phantoms, using larger illumination area, employing two wavelengths, and/or gathering more knowledge of the surrounding tissue by 3D imaging. Other artefacts that are observed in the UOT images are the darker regions just under the inclusions. These shadows may be caused by the higher absorption of the inclusions resulting in a smaller number of photons underneath it. Additionally, variations may also arise due to inhomogeneities in the agar phantoms. Typical US artefacts like enhancement and shadowing could also affect the final results of UOT. As seen from US images in Supplementary Fig. S3, it does not seem to be the case in the current measurements. However, if that was the case in the future measurements it could be accounted for by analysing the US images, since they are also being taken during the UOT.

## Conclusions

In conclusion, by using ultrasound optical tomography equipped with a Tm^3+^:LiNbO_3_ spectral hole burning filter, we have demonstrated the ability to image tissue-mimicking phantoms with inclusions having the absorption and reduced scattering coefficients that match those of real human breast tissues and tumours. The imaged agar-intralipid phantoms were 5 cm thick and had 12 × 12 × 12 mm size inclusions positioned in the middle, i.e. at 2.5 cm depth. The inclusions were clearly visible in all six UOT images, although some shadows and large-scale variations were also observed. The latter ones were probably caused by the difficulties in precisely positioning the ultrasound transducer and aligning it with the lightguide, which collected the signal. In the future, this could be prevented by using a specially designed more precise transducer holder. As for in vivo applications, approaches not relying on the physical blank phantoms could be employed, for example, the use of digital blank phantoms, two-wavelength experimental set-up, increasing illumination area, 3D imaging.

The calculated Contrast-to-Noise-Ratio (CNR) values exceeded 1 in all cases but were characterized by significant errors due to the aforementioned inhomogeneities in the images. Still, CNR values do seem to increase with the absorption coefficient ratios and possibly also with reduced scattering coefficient, although this is even more uncertain. However, the UOT signal does seem more sensitive to the changes in absorption than to changes in reduced scattering. The main drawback of the UOT technique in this experiment was the comparably long image acquisition time, which was nearly 9 min. Since this was mainly caused by the fluorescence to the lowest excited Stark level in Tm^3+^:LiNbO_3_ filter, this issue could be solved by using a crystal filer where the lowest ground state crystal field Stark levels is spectrally well separated from the other crystal field Stark levels.

The excellent contrast demonstrated between the realistic tumour-like inclusions and the healthy breast tissue mimicking part of the phantoms suggests that UOT has a great potential for medical imaging. Since changes in optical properties are not detected by conventional clinical imaging techniques the information provided by UOT is complementary and could provide useful information. These possibilities strongly stimulate further work on exploring the UOT technique’s capabilities for clinical use in in vivo settings.

## Supplementary Information

Below is the link to the electronic supplementary material.

Supplementary Material 1.

## Data Availability

Data can be provided upon a reasonable request from the corresponding authors.
