# Supplementary material for: Characterization of tumour-like inclusions in breast-mimicking phantoms using ultrasound optical tomography
Source: Sci Rep. 2025 Sep 15;15:32543. doi: 10.1038/s41598-025-18902-1 (PMC12436635; doi:10.1038/s41598-025-18902-1)

Characterization of tumour-like inclusions in breast-mimicking phantoms using ultrasound optical tomography

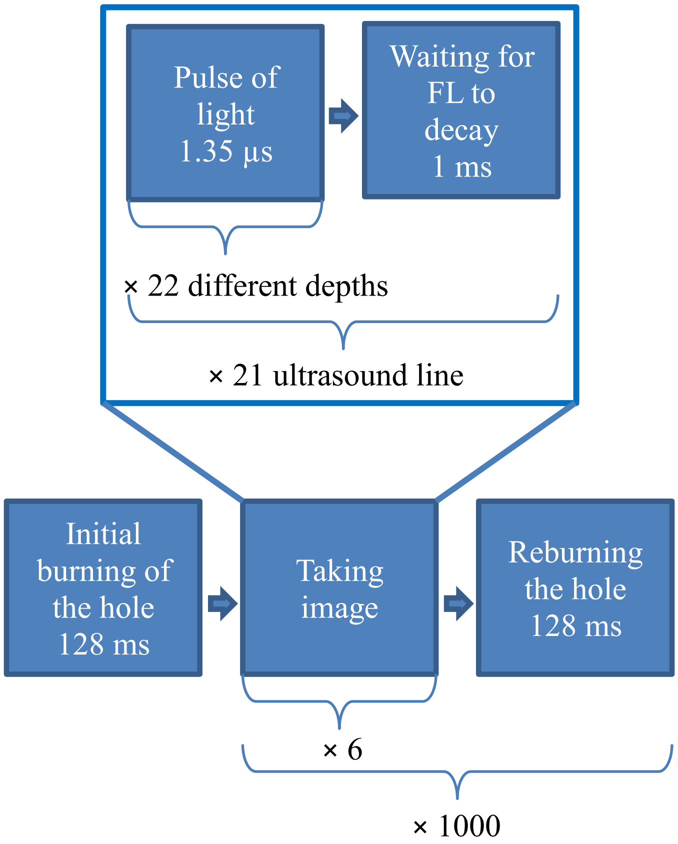

Fig. S1. Schematic representation of image acquisition steps. The durations of each step are presented within the block. First, the spectral hole is created (burned) in the filter. Then 22 subsequent pulses of light are sent in as the ultrasound pulse propagates through the phantom in order to image pixels at different depths (z). Afterwards a wait time of 1 ms is added to wait for the fluorescence (FL) to decay. To get the image, the sequence is repeated for each of the 21 parallel ultrasound lines. After 6 images are taken, the hole has to be recreated (reburned). This sequence is repeated 1000 times which results in total 6000 averages.

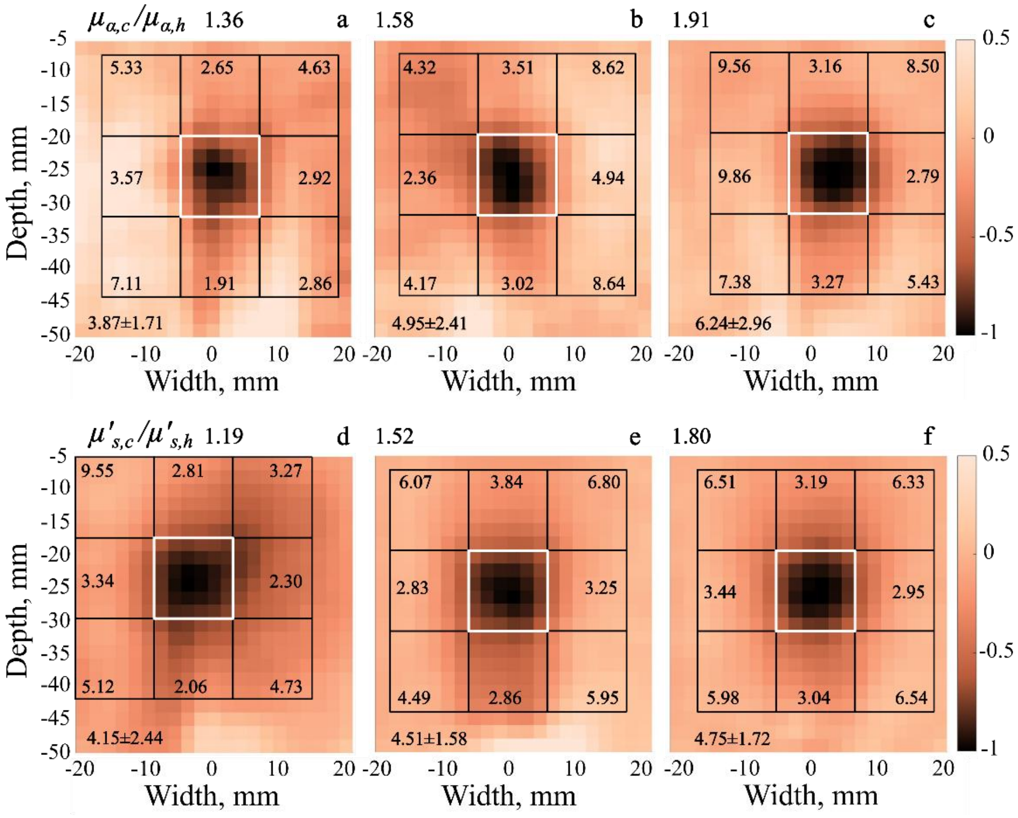

Fig. S2. UOT images of 50 mm thick agar-intralipid phantoms with tumour mimicking inclusions and the calculated CNR values. (a), (b), and (c) show the images with increasing inclusion absorption; (d), (e), and (f) show the images with increasing inclusion reduced scattering. Absorption and reduced scattering coefficient ratios between the inclusion and the background ($\mu_{\alpha,c}/\mu_{\alpha,h}$ and $\mu'_{s,c}/\mu'_{s,h}$, respectively) are indicated above each subfigure. All images are normalized such that the value of the darkest pixel is -1. The white and black square-shaped contours indicate Regions of Interest (ROI) for the signal and background, respectively. The number in each background ROI indicates the CNRs of the region. The average values of the background CNRs and their standard deviations are presented at the bottom left corner of each image.

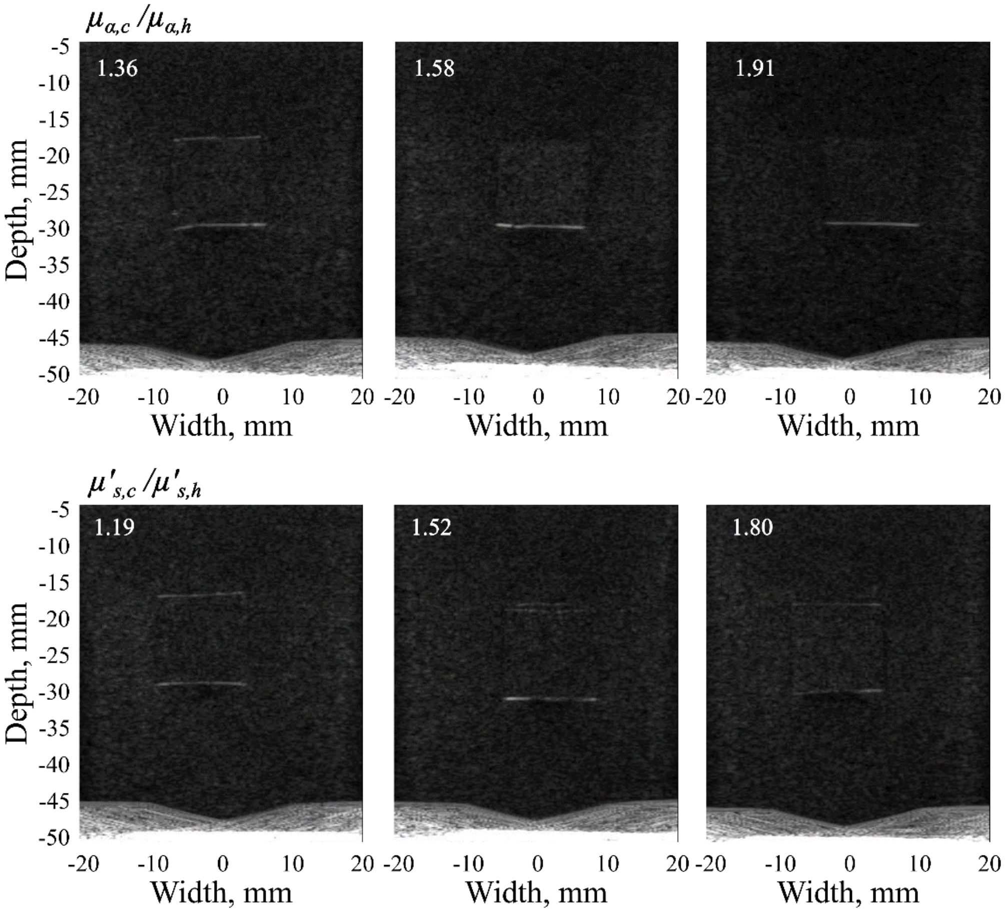

Fig. S3. Ultrasound (US) images of all the phantoms in the Bright (B-) mode. Collections of small air bubbles at the borders between the inclusions and the surrounding background phantom material show up as thin white lines in the images.

Table S1. The RGB values for five colors used for the UOT image's color scheme:

R:	0	185	246	255	255
G:	0	92	144	199	230
B:	0	60	108	160	214

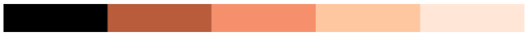

Supplement: Supplementary file 1 — Supplementary Material 1. [file 41598_2025_18902_MOESM1_ESM.pdf]
